# Parental perspectives on school attendance problems and the role of municipal support systems

**DOI:** 10.3389/frcha.2025.1589988

**Published:** 2025-06-09

**Authors:** Ulla Irene Hansen, Ellen Kathrine Munkhaugen, Kenneth Larsen

**Affiliations:** ^1^ Department of Education Science, University of South East of Norway, Horten, Norway; ^2^ Division of Mental Health and Addiction, Oslo University Hospital, Oslo, Norway

**Keywords:** parents' perspective, collaboration, empowerment, knowledge, school attendance problems

## Abstract

**Introduction:**

This study aims to explore parents’ experiences collaborating with support systems in a Norwegian municipality for children who experience school attendance problems (SAP). The heterogeneity of SAP highlights the need for individualized measures tailored to each student's needs and collaboration between schools, municipal support systems, and the Child and Adolescent Psychiatric Outpatient Clinic (BUP). Empowering parents to advocate for their children is crucial in managing challenging situations.

**Method:**

In total, 11 parents of students who received support from a school absenteeism team (SAT) participated in the study. This qualitative study uses focus group interviews as a data collection method.

**Results:**

The results underscore the complexity of addressing SAP within municipal support systems and the need for a dedicated SAT to support students and empower parents. The parents underline the importance of having collaborative practices, evidence-based knowledge, structures, and procedures to ensure interventions are relevant and predictable. They emphasized the importance of establishing collaborative practices within the municipality.

**Conclusion:**

Collaborating with parents as equal partners increased the sense of wellbeing for the parents and enhanced the students’ decision-making process.

## Introduction

School attendance problems (SAP) comprise different types of absenteeism, such as anxiety-based school refusal (1), truancy (2), and parent- and school-based school withdrawal (3). Both school attendance problems and school absenteeism are problematic because of their frequency and/or duration (4). Students with SAP have a significantly increased risk of high school dropout, leading to potential unemployment, poverty, and social isolation in adulthood. SAP is a phenomenon that faces a complex and heterogeneous symptom presentation (5, 6). Various individual, school, and family factors influence this multifaceted issue. Individual factors encompass psychological problems such as anxiety and depression, learning difficulties, and neurodevelopmental disorders, such as attention deficit hyperactivity disorder (ADHD) and autism spectrum disorder (ASD) (7, 8). School factors include, for example, the school environment, teacher–student relationships, peer relations, and bullying. Havik et al. (9) found an association between school refusal and truancy-related absenteeism and teachers’ classroom management in lower secondary school. They suggest that one factor that may increase the risk of school refusal and truancy is poor support from teachers. Transitions between education can be challenging for students with SAP and special needs (9). Ingul et al. (10) underline the importance of identifying transition problems in lower secondary school. Family factors include health issues, family dynamics, economic challenges, and difficulties within school-home collaboration (11, 12). Addressing all these factors requires a systemic approach involving schools and municipal support services to implement customized strategies that improve school attendance and prevent chronic absenteeism (12–14).

Parents of children who exhibit school attendance problems and children with special needs are vulnerable, often experiencing stress on multiple levels, which impacts their overall wellbeing (15). Parents support and influence their children's development, and being a part of their children's lives is a lifelong commitment (15–18). The complexity of parenting children with special needs can be overwhelming, and parents are often deeply involved in various aspects of their child's education (19). Acknowledging the parents’ multifaceted role as part of the ecological system is essential to understanding their role. Thus, empowering parents is beneficial in addressing SAP. Empowerment is an internal process through which individuals gain control over their lives and ensure democratic participation within the community. This process highlights the strengths and competencies of each participant in the ecological system (20). Professionals are responsible for listening to parents who advocate for their children, recognizing that parents are constantly involved in their children's lives and often best positioned to understand their needs (17, 21–23).

Due to the multifaceted issues associated with SAP, Bronfenbrenner's Ecological Systems Theory (24) offers a comprehensive framework to analyze and address severe SAP. This theory elucidates the different environmental contexts and describes developments influenced by the interaction between these contexts. It emphasizes the importance of examining the interplay between the child and their family, school, and community to identify factors that may affect their school attendance. It also highlights the need for broader support when a student is absent from school (24). SAP is a multifaceted problem where complex school- and community-based issues may contribute. The arguments for implementing frameworks such as multi-tiered systems of support (MTSS) focus on how the different contexts impact SAP and problematic school absenteeism. The student's wellbeing is linked to a healthy school environment and well-functioning community systems. Therefore, a systemic approach such as ecological theory may combine individual and contextual factors (14, 16, 25, 26).

“Hemmasittare och vägen tillbaka” is a Swedish multimodal and manual-based program (henceforth referred to as HSP) designed to increase school attendance and reduce anxiety, depression, and other psychiatric symptoms (27). HSP was developed in Sweden, based on cognitive behavioral therapy (CBT), and has been developed in an environmental context. This multisystemic framework incorporates academic, behavioral, and social-emotional support based on the students’ needs. HSP is organized into three phases: (1) the assessment phase, (2) the intervention phase, and (3) the maintenance phase. Implementation of HSP or other systemic, multimodular intervention frameworks for SAP in municipalities often requires a school absenteeism team (SAT) with the following competencies:

1) The assessment phase is ongoing throughout the whole intervention period. The initial assessment phase is a core element of HSP, assessing the cause and reasons for absenteeism through assessment tools, school reports, and reports from other relevant support systems. The key part of the initial phase is to establish a safe and trustworthy relationship between the HSP professional, the student, and their parents, and get an overview of the reasons for the student's attendance problems. Strengthening the collaboration between the home and school is one of the key factors to address, alongside the support system around the student, combining all the measures that can help the student. Several services are relevant when children experience school attendance challenges, such as the Educational and Psychological Counseling Service (PPT), the Child and Adolescent Psychiatry Outpatient Clinic (BUP), the Norwegian Welfare and Labor Administration (NAV), and the Child Welfare Services (BVT).
2) In the intervention phase, the SAT professional will plan the student's activity. This plan may include behavioral interventions, academic adaptations, and individualized psychological interventions, and it is always based on the assessment that is verified by the student and the parents before implementing the plan. The plan is evaluated weekly by the student, their parents, schools, and other relevant professionals, and changes may be made to accommodate the student's needs. When the student has psychological symptoms, such as anxiety and depression, it will be necessary to consult and refer to, for example, the BUP. The SAT professional has been trained to understand this complex phenomenon, but it is important to get support from a specialist if needed. During this phase, the SAT professional will still be the closest to the family. Still, sharing knowledge about the student's needs and the measures implemented to enhance the relationship with the school and the PPT and prepare the student for the next phase is necessary. Once a deeper understanding of the student's needs and individualized measures has been established, the SAT professional will gradually transfer the responsibility for the student to the school and the PPT in the municipality.
3) The SAT will still be available in the maintenance phase but will be more distant. The SAT professional will still be available in this phase as a supervisor for the school and the support services, but the school will now be responsible for the activity plan and support at school. There can still be some support from SAT professionals, but the student and their parents must be willing to trust and accept support from other professionals as well.

The estimated duration of HSP is approximately 1 year. Still, the SAT noted that it would need more time for some students, especially when the student has been absent for an extended period and has developed a sense of mistrust in, and alienation from, the system. We aimed to explore parents’ experiences of collaborating with municipal support systems to address SAP using a systemic, multimodular intervention framework implemented by the municipality's SAT. This framework presented in this study represents a holistic approach that ensures a comprehensive assessment of the underlying causes of SAP and facilitates targeted interventions to address students’ specific needs. A core framework element is helping parents and the school system understand and address absenteeism. The framework captures the need for coordination and collaboration between the support systems when handling this multifaceted issue.

The heterogeneity among students displaying SAP highlights the need for comprehensive intervention strategies. Multimodal treatments, based on thorough assessments and involving interdisciplinary approaches, are recommended for their effectiveness (2, 28). It is crucial for municipal support systems to acknowledge the overlap in psychiatric symptoms across types of absenteeism (2). Identifying individual and organizational factors that increase school attendance is essential to improve the utility of interventions for all students (12, 29). Previous research has emphasized that understanding the heterogeneity of this phenomenon is essential for understanding the student's situation, interests, and needs and for planning effective interventions. These interventions should be pedagogically sound, evidence-based, and codeveloped with the student, their family, and the school (12, 14, 30). Pérez-Marco et al. (4) conducted a systematic review of intervention programs for SAP. The study aimed to systematically review programs designed to reduce or prevent school refusal and truancy. They underline the fact that SAP affects family, peers, and teachers; therefore, it is essential to address SAP intervention in the context and implement a simultaneous intervention in the family. CBT strategies used in school refusal are evident, and truancy programs, such as psychoeducational techniques to better understand the student's situation, are preferred. Effective programs are characterized by cooperation and collaboration between all participants (4).

Due to the multifaceted problems of SAP, effective intervention programs are characterized by interdisciplinary perspectives so that students’ treatment needs are met efficiently (4) and there is collaboration and cooperation with all relevant participants in the systems (4). In addition, there is a consensus that early identification and intervention are crucial to prevent severe school attendance problems. For example, Kearney et al. (14) suggest that an MTSS model can be used as a framework to prevent and detect attendance problems early and intervene in and support students experiencing SAP. Effective interventions are crucial when students show signs of absenteeism. Teachers need the knowledge to identify at-risk students and prevent severe school attendance problems (10, 31). Few studies report parents’ perspectives on adequate municipal support of students with SAP.

In Norway, students up to the age of 24 have a legal right to education. The first 10 years of schooling are mandatory, while the final 3 years are voluntary. The Norwegian Education Act emphasizes the responsibility of schools to collaborate with parents to facilitate meeting students’ special needs and monitor and implement measures to address student absenteeism. Norway has no national registration system to monitor absenteeism among Norwegian students.

### The present study

Severe SAP is a complex, global issue that has received increased attention recently. However, the literature regarding the parental perspective needs to be more extensive, especially concerning the topic of municipal-level support. There has been limited research on how a municipality-based support system may organize its support and what elements are needed in this support to give students and their parents sufficient help. Studying parents’ collaboration experiences with schools and municipal support systems is required to understand how to address the complexity of this phenomenon. The impact of collaborative efforts among professionals in municipal support systems needs further exploration.

This study's objective is to explore parents’ perspectives on interacting with the municipal support system. Further information that may enhance municipal-level support and the implementation of HSP.

## Methods

### Participants

The study targeted the parents of 11 students who had received services from the SAT in a rural municipality during the 2021–2022 and 2022–2023 school years. The SAT facilitated recruitment by sending written invitations and information packets to the students’ parents, seeking their consent for participation. Of the 11 families contacted, nine agreed to participate.

Regarding the participants, six were mothers and three were fathers. One mother was single, and one student had divorced parents. The parents’ employment varied in percentage, and they reported having an agreement in place with their employer, adapted to their situation and their child's mental health severity, in combination with full financial support from NAV. The student cohort represented in the study displayed varied patterns of absenteeism; the mean percentage of absenteeism was 70%. These patterns varied within the continuum from refusal, with 50% attendance, to 100% absenteeism from school, confirmed by the SAT professional in collaboration with the school system. One student was diagnosed with Tourette's syndrome, two with ASD, one with ADHD, one with separation anxiety, and one with no verified diagnosis. No instances of bullying were reported in this sample.

This qualitative study employed focus group interviews as the data collection method. The rationale for using focus group interviews stems from the phenomenological approach in this study and their ability to elicit rich, interactive discussions, generating valuable data. The benefit of gathering data through focus groups is the dynamic that occurs in interaction when participants reflect together and bring several perspectives to the table. As defined by Morgan (32), focus group interviews have three key aspects: they are a method specifically for data generation, group interaction is a crucial data source, and the researcher plays an active role in facilitating discussions to generate data. The method typically involves structured discussions among 6 to 10 participants who are often strangers, creating a dynamic conducive to diverse viewpoints and rich data collection (32–34).

The focus group session in this study lasted approximately 2 h and was conducted by authors one and two. In the first minutes of the interview, the parents and authors introduced themselves and had some “warm-up” conversations facilitated by the authors. The authors then explained how the interview would be conducted. Author one introduced each question. The questions to be discussed were supposed to last approximately 20 min each. The questions were presented as open-ended questions about their situation and about the support they received from the school and the municipal support system. They discussed their circumstances before the SAT was implemented and their experience of being assigned an SAT professional. They were also asked to discuss the collaboration between the school, the PPT, the BUP, and other relevant support services. They were asked about how an SAT could contribute to their situation and their opinion on the key factors for an SAT to implement, involving evidence-based knowledge, collaboration, individual plans for the student, and organizing factors. The researchers developed the questions based on previous research. The participants received feedback on the results in a parental meeting after the data analysis had been completed and discussed.

### Procedures

A rural Norwegian municipality has established an SAT to strengthen student support. The team's goal is to initiate measures that improve school attendance among primary and lower secondary school students who exhibit school absenteeism for more than 10% of the school year. Identifying the interventions and organizational dynamics that promote increased attendance is crucial for informing the support system around these students. The complexity of these efforts may intensify for students approaching the end of lower secondary school and preparing to transition to high school or vocational training programs.

The HSP framework is comparable to the Norwegian context. The transition between countries with similar structures, norms, and values may increase the success of an adaptation to a local context (35–37). The SAT is organized in the municipality to support children, families, schools, and other related services within the municipality and neighboring systems. Principals at the schools in the municipality refer to the SAT about a student's absenteeism after initial measures have been conducted. SAT professionals work individually with students and parents. An HSP framework requires a strong relationship with the student that can manage the difficulties that may arise when measures are implemented. In this initial phase, the student is referred to the PPT because it is important to establish a rationale for the resources required to support the student in such a situation. This is a new practice in this municipality because a student with no academic difficulties is not typically referred to the PPT. The SAT professional is the “key stakeholder,” organizing the collaboration with the school, parents, students, and other relevant professionals in the municipality, the PPT, and the BUP. The SAT meets weekly to discuss students and measures, deciding if other professionals can assist. Furthermore, it regularly receives supervision from trained professionals.

This study is a part of a larger project named “Back to School,” and was approved by the Regional Ethics Committee with application ID 234850 and adhered to the data protection guidelines stipulated by Oslo University Hospital. Ethical considerations also extended to ensuring the anonymity of the participating children and parents, aligning with the National Committee for Research Ethics in the Social Sciences and the Humanities (NESH) recommendations. The study's alignment with the Personal Data Act and the Declaration of Helsinki was overseen by municipal legal authorities. The participants’ demographics are sensitive data and are therefore not described in detail.

In addition, the first author's role as a supervisor for the SAT and the inclusion of coauthors in the study design served to mitigate potential biases, such as the close relationship between the first author and the SAT. This upheld the integrity of the research process. Authors one and two supervised the SAT during the implementation phase of HSP. The authors were educated in the HSP framework and trained as supervisors. Author three has experience in schools and specialist healthcare for children with ASD. Limitations are discussed in the Conclusion section.

### Data analysis

Constructivism was the theoretical underpinning of this study. The data analysis followed a phenomenological approach, guided by Braun and Clarke's (38) six-phase thematic analysis method. This approach allowed for an in-depth exploration of patterns and themes relevant to the study's objectives. The initial phase involved transcription by the AI tool Whisper (AI = artificial intelligence), and then the first author checked the transcription to verify that the transcription was correct. All the authors then read each interview comprehensively to grasp the overall narrative and identify keywords and patterns. In the first inductive exploration, quotations related to the research questions were sorted into categories by all the authors to identify patterns and themes in the data. To look for patterns, the quotations were marked with colors, and their use in this manuscript illustrates how they relate to the themes. This was another approach that increased the validity of the results.

In the next step, the meaning-carrying units were abstracted into codes as part of the content analyses, and the codes were then sorted into subcategories. In a few cases, the meaning-carrying units needed to be recoded, resulting in another subcategory. All the authors assessed the coherence and validity of these themes together. Tentative codes were discussed and revised until a consensus was reached. The authors engaged in ongoing reflexive practice to acknowledge any biases and assumptions that could influence the results. The authors then met both physically and online. The authors met several times to examine relationships between codes, themes, and subthemes. The authors searched for previous research in this field to validate the themes. The quotations used in this manuscript show how the data were generated and the subsequent conclusions. To make this process valid and increase the coding process's consistency, being a part of a research group is helpful. In this study, there were three researchers in the group to ensure that the coding and interpretations illustrate the data. The results were discussed in relation to previous literature in the field to strengthen the study's validity. This study is small, so validating the results through previous research is important. The final phase involved defining and naming the themes for the presentation (38). The analysis was conducted manually, resulting in the emergence of three main themes.

## Results

This research explored parents’ experiences with collaboration with schools and local support systems, such as PPT and BUP, for students facing significant refusal and SAP. The main themes are as follows: (1) barriers to effective support, (2) professionals’ knowledge, and (3) collaboration. The parents supported each other, and their verbal and non-verbal responses created a safe environment and inspired the participants to share. The relief at finally understanding that they were not alone in this situation was a euphoric experience for the parents and may have strengthened their participation.

### Barriers to effective support

This main theme offers insights into parents’ experiences before the involvement of the SAT. It encapsulates the parents’ feelings regarding their child's absenteeism and their need for support when advocating for their child.

#### A sense of being left alone

The participants consistently emphasized the importance of peer support among parents in enhancing wellbeing and mitigating the adverse effects of facing the challenges on their own. One participant stated, “I wish I'd known others in the same situation…Someone to discuss and reflect with. Because you think you're the only one.”

Sharing parental advice and personal experiences greatly enhances the understanding of individual situations, empowering parents to better navigate different systems and opportunities. Exchanging experiences in a safe environment fosters a sense of community. This revelation has significant implications for municipal support mechanisms and highlights the benefits of facilitating connections among individuals facing similar challenges.

Prior to the SAT's involvement, the parents reflected on their circumstances.

The conversation was as follows. One parent stated, “…[L]oneliness is the first thing that springs to mind…” Another parent said, “Yes, I was completely at a loss…,” followed by, “A sense of despair and an inability to influence the system….”

In the other focus group, one parent said, “In our case, somatic illness is the reason why it is hard to attend school … and if my child is at the hospital, they will get support… but if my child is at home and cannot attend school, nobody helps.”

One family had to leave and move to another municipality to access effective measures. They said, “The old school put all the responsibility (for non-attendance) on us….”

Before the SAT was established, the parents felt solely responsible for implementing the recommended strategies without thorough evaluation or oversight by professionals. This stemmed from a lack of mandated supportive policies at home, making the parents the primary source of support for their children and with little support from professionals. The parents expressed a sense of working in isolation and a strong commitment to following the recommendations of educational professionals to ensure their children attended school.

One of them said, “You have to force your child and try to persuade them. And when they're small, you can carry them to school and hope that this period will pass, although you can't do this for an extended period, but it's hard to know what to do.” Despite feeling ignored and blamed, the parents also sensed that the system had expectations, highlighting a conflicting role that needs attention, as illustrated by the following statement by a parent, “I'm thinking about all the conflicts I had with my child when they had a tough time because I felt obligated to get them to school.” Another parent said, “And the worst is that ultimately, you begin to seriously doubt your own parenting skills.”

Several parents reported using physical methods, such as forcing their child to attend school and ensuring they stayed there, in line with the guidance of educational professionals. This often coincided with a sense of deep despair and sorrow.

#### Being heard—parents as experts

This section describes the parents' experiences and their role in decision-making.

The conversation was as follows. One parent said, “It took a long time before we were listened to…I think if the school had listened to us earlier, it would not have become so serious….” Another parent in the group stated, “We felt it differently. The school listened to us, but they had no solutions.” One parent replied, “…It took a long time before we were heard, and when we were heard, the damage had already been done….”

These statements highlight the importance of including parental perspectives to enhance a child's support system. Some participants noted that their schools were supportive and encouraged parental involvement in collaborative efforts. However, these schools often lacked the necessary skills to support the child. A common theme among several parents was the delay in early identification and intervention for school attendance problems due to a systemic lack of knowledge and a policy framework. Various factors, including the undervaluation of parental knowledge, further compounded this issue.

Parents expressed frustration due to feeling excluded, unacknowledged, and being expected to ensure regular school attendance. They pointed to a widespread lack of understanding about the causes and perpetuating factors of school attendance problems in schools, the PPT and the BUP, often characterized by subjective judgments and opinions. This lack of objective understanding, frequently replaced by personal biases and attitudes, prevented them from effectively encouraging consistent school attendance. Recognizing this barrier is crucial for implementing holistic strategies to address school absenteeism. This knowledge gap undermines the efforts of parents to promote school attendance, thereby impeding the development of a comprehensive understanding of SAT problems and creating effective interventions. One parent said, “I thought every time new professionals showed up… maybe this will work because no one knows where to begin.” Further, another parent said, “You try so hard, teachers and people around you give you advice… I know there are many problems we need to address and many services that provide help, but it’s challenging to find something that works.”

The participants reflected on their parental authority, highlighting the marginalization of their experiential knowledge and expertise about their child within a system that ostensibly relies on their involvement. They felt that the intervention strategies were primarily based on the subjective judgments of professionals rather than on empirically grounded knowledge or parental input. Despite these challenges, their confidence in the support systems remained in place, bolstered by the introduction of new methodologies or the involvement of new experts.

#### A sense of distrust

This section contains parental experiences of the system regarding their role in collaboration with professionals.

One parent said, “…What are we doing wrong? What am I doing wrong when we cannot get our child to attend school? You begin to blame yourself and your partner, so to speak…this situation negatively impacts the family.”

Another parent continued, “The situation gets all-consuming…. from the time our child goes to bed or doesn't want to go to bed because he dreads going to school. Day after day…around the clock.” Another parent elaborated, “If you're not engaged physically with your child's attendance, your brain is constantly thinking about what you can do better and what will happen tomorrow.”

The participants described their parenting experiences as being under constant scrutiny, with frequent questioning of their parental abilities by professionals. A significant disconnect between the parents and the professional system was evident throughout the discussions, reducing confidence in the system's efficacy and integrity. One parent said, “It's my collaboration with the BUP that gives me the strongest sense of distrust. You leave the place, and you just scream, you feel like a guilty person, it's horrible.” Parents reported a perceived lack of knowledge and established protocols, which prevented them from effectively advocating for their child's educational needs. This was linked, for example, to delays in obtaining evaluations and reports from the BUP and the PPT, which they believed were crucial for ensuring the necessary educational support. When these evaluations and reports were finally received, they were often outdated. This left the parents feeling that the situation was highly disorganized. The resulting distrust toward the municipal education system, including schools and teaching staff, was stressful for the parents. Attempts to seek help from external experts often failed to address these issues, as these experts frequently had no understanding of the severity and urgency of the crises the students and their families were facing. This left the parents feeling isolated. The parents expressed a sense of distrust concerning their abilities as parents while also perceiving a burden of responsibility and blame, which negatively impacted their psychological and emotional wellbeing. Understanding this phenomenon is crucial for raising awareness and developing a support infrastructure that appropriately involves parents.

### Professionals’ knowledge

#### Pathways to better care

This theme highlights the importance of understanding parents' and children's needs, focusing on the implementation of holistic support by the SAT. This section highlights the parents' reflections on their experience after the SAT became involved.

The participants were eager to share their experiences, and one said, “… I felt safe when the SAT was involved.” One parent stated, “Yeah, my child had similar feelings. Now my child has a person who helps to handle their frustrations about the school,” and continued, “The SAT met my child at the school several times during the week. And if they didn't manage to attend school, they talked on the phone or at our house.”

Another parent stated, “The SAT approach was sensitive …That was important for us, and to be sure that it was the right person… It's crucial that the person doesn't just burst in.” Another parent added, “I was pleased with the method the SAT professional used. It was okay to take it slowly, to take it one step at a time.” One parent continued, “The SAT professional had a very trust-building personality.” Another parent said, “I agree. Still, the turning point for us was when I met one (an SAT professional) who understood our situation completely; that was fantastic.”

Professional consultation and the perception of equal interactions have therapeutic benefits. One parent said, “Having one person that could take all issues with the school was a relief.” Another parent replied,

Wow, suddenly, we didn't have so many conflicts at home. We could relax a bit, and we're not that stressed anymore. The measures carried out were more individualized, and the mother-son relationship that you are so independent of when you're going through difficult things improved.

One parent stated, “I think every municipality should have such a team because they meet our child and us and put individualized measure in practice.”

Providing practical support, such as respite for parents through child engagement activities, strategic interventions, and advocacy efforts, significantly enhances family welfare. This approach reduces both psychological and practical stress. The parents emphasized the importance of the SAT's accessibility, highlighting how easily it can be accessed in times of need. The parents emphasized the importance of the SAT's initiatives, knowledge base, and methodological frameworks. They found the collaboration between the SAT, the students, and themselves beneficial. They appreciated the SAT's ability to identify and address student absenteeism issues, valuing its advocacy for their concerns and its role in alleviating their guilt. In addition, the SAT's ability to integrate parental insights into customized and acceptable plans empowered the parents. One parent stated, “The school was okay, supportive and positive, but they struggled. They should learn about school attendance problems like we do now because it's so great when you realize that a measure is ‘to the point’ and works….”

The parents noted that the effectiveness of professionals in improving attendance depends on their willingness to make an extra effort. Having someone to talk to during difficult times at home, and professionals willing to “go the extra mile” was very important to the parents. This kind of support is crucial for fostering confidence and trust. A consistent “parental voice” emerges, characterized by a sense of relief and the alleviation of a significant burden. Building trust among families and relevant professionals, such as teachers and the PPT, is essential for the effectiveness of these interventions. In addition, a fundamental understanding of absenteeism is seen as a crucial prerequisite for such interventions to promote attendance at school effectively.

### Collaboration

#### Coherence in educational and health services

This section contains the parents’ perceptions of access to services and the service's effectiveness in cross-agency and interdisciplinary collaboration. The parents shared their views on the system's collaboration regarding assessments and initiating measures to support their child's situation. They also emphasize the need to advocate for their child.

One parent said, “You must be your own lawyer and find all the available knowledge. There's more advice in Facebook groups.”

The parents reported difficulties navigating the various services available, noting that problematic school attendance problems increased the need for access to diverse support systems. They also perceived consistent school attendance as a prerequisite for activating these support mechanisms. One parent said, “We didn't make any individual education plan (IEP) because the schools can't do much these days….”

The parents lacked a comprehensive understanding of the various components of the municipal services, their interrelationships, and the connection between the municipal support system and special health services such as the BUP. Parental awareness and understanding of the support system appeared limited. Navigating these complex systems was challenging, leading parents to rely on a single professional in the SAT.

One parent said, “Our SAT professional refers to a counselor at the PPT…I guess they talk with each other, so I think it works well.”

When a child exhibits school attendance problems, parents often encounter a lack of systematic procedures and protocols for intersystem collaboration to improve school attendance. This lack of structured collaborative frameworks can lead to exclusionary practices, especially without strategic coordination within the system. In addition, the parents found the organization of different services in the municipality, the BUP, and the PPT to be fragmented. The services were inaccessible, and the referral process was demanding. Significant time passed before the parents received support from the municipal system and the BUP.

The parents felt that the efficacy of professional support is influenced by the professional’s expertise, personal attributes, and commitment to going beyond the basic requirements, which is characterized by a willingness to offer additional assistance and support. They expressed uncertainty about whether referring children to other services with long waiting lists was an effective solution for addressing mental health issues resulting from absenteeism.

One parent said, “… [H]ow mentally ill do you have to be to get access to the BUP?”

The parents identified another bottleneck in the system: the BUP's role as a diagnostic authority and its recommendations for financial support from NAV. One parent stated, “This was the hardest time. My child went through the third diagnostic process, was not attending school, and was afraid of being left alone at home; at that time, you had no right to financial support… your child must have a diagnosis, and that takes a year.”

The participants in the study identified specific shortcomings in the educational infrastructure and resources, particularly in allocating essential resources for school attendance problems. This disconnect between schools and students negatively impacts parents, often burdening them with the responsibility of ensuring their child attends school and managing the complexities of their situation. This added responsibility posed significant challenges. The parents reported that navigating these systems was extremely burdensome, adding to their demanding circumstances. This significantly affected their own professional lives, as they had to support their child and interact with various support systems. Some of the parents worked part-time, while others could not work due to their child's need for care. One parent said, “I'm working part-time, and my employer is very understanding so that I can adapt my work schedule to the needs of my child….”

Various strategies were employed to address these issues, including administrative measures, financial support from NAV, and medical leave provided by general practitioners (GPs). One parent added, “…There's a lot of work: meetings, emails, telephone calls… I've never felt so tired before.”

The parents described a significant lack of knowledge in adapting educational environments to create a secure and predictable setting that meets the needs of their children with neurodevelopmental disorders and mental illnesses. One parent stated,

My child needs to wear a hood, and when they attended a cooking class after a long period of absenteeism, the teacher told them to remove the hood for hygienic reasons …and my child came home and told me this. The teacher should never have said that to my child… but now it's too late.

From the parents’ perspective, the lack of integration and collaboration between schools, the PPT, and the BUP was detrimental to them and their children. They described seeking help as a struggle and attributed the barriers to effective intervention for absenteeism and the successful reintegration of students with SAP to failures in systemic collaboration and deficiencies in the BUP's support mechanisms. The parents emphasized the importance of knowledge in organizing municipal support and counseling systems to meet the needs of their children. They suggested that a better understanding of the system is crucial for developing comprehensive strategies to address the complex issues surrounding SAP.

## Discussion

This study explores parents’ perspectives to provide knowledge that may address municipal-based practices.

The parents in the present study frequently experienced distrust in the system, feeling their voices and concerns were not adequately heard or valued. This distrust was associated with disempowerment and a strong sense of being disconnected from the system, as they described feeling alone and helpless. The parents felt sidelined in decision-making processes related to their child's education and wellbeing and, at the same time, felt obligated to support the school in their efforts to tailor measures for their child in school. This conflict must be addressed. In line with other studies, an in-depth understanding of what children need and the importance of parents’ perspectives for individualizing measures have been highlighted (12, 19, 22). The parents in this study also underscored the importance of mutual trust in every part of the system as a key factor in therapeutic relationships that may result in positive outcomes. A lack of empowerment can negatively impact the effectiveness of support measures. Gray et al. (19), who studied 12 students with ASD who had experienced school refusal, found that strengthening the partnerships between parents and schools can positively impact autistic students’ experience of school. Hsiao et al. (22) concluded that parent involvement is a key factor in school success in their guidelines for empowering parents. This aligns with previous research that shows the importance of collaborating with parents to support a student with SAP (17, 19, 22, 39).

Another significant barrier identified in this study is the perceived lack of understanding among professionals about the complexities of school absenteeism. The parents reported that this lack of understanding resulted in inadequate or inappropriate responses to absenteeism issues. This increased absenteeism and exacerbated the problems for most of them. They noted that insufficient knowledge, poor systematic intervention, and delayed measures contributed to ongoing absenteeism. Furthermore, the parents expressed that their children did not receive a proper education because of their absence from school, and the teachers were not familiar with working with students at home. They reported that this lack of flexible, responsive, and tailored measures may be a significant barrier to meeting their children's educational needs. The measures adopted often lack flexibility, which negatively impacts the students’ sense of autonomy. In line with other studies, this study's results highlighted that the parents wanted to ensure that the students' voices were included in important questions about their situation (19, 20). Studies have shown that one effect of SAP is that students frequently lose the chance to discuss their unique situations and articulate their needs (15, 40).

Further, the parents expressed that the system tends to underplay their crucial role in addressing their child's absenteeism. This impacts collaboration with parents, which is crucial for developing effective and sustainable intervention strategies. This is also underlined by Preece and Howley (15), who studied the impact of school refusal and ASD on students and their families. Undervaluing parents’ insight and active participation leads to a disconnection between the support provided and the family's actual needs. The complexity of parenting children with absenteeism is more challenging than parenting children who do not experience such challenges. The school, the PPT, and the BUP need to pay more attention to parents and make them feel heard and cared for (17, 19, 39). Addressing these challenges is essential for creating effective strategies to reduce SAP and to ensure that the support system effectively meets the needs of students and their families (15, 39).

The parents in this present study agreed that the support offered by the school, the PPT, and the BUP was often characterized by inconsistencies, fragmented efforts, and a lack of coherent strategies across different levels of schools and support systems. They reflected on the difficulty of getting the system's help when needed. Their children suffered from mental health issues, such as depression, anxiety, and trauma. These mental health issues were often trivialized and attributed to parenting. This frequently led to despair among the parents. However, some parents reported that the school responded positively to the student's needs, often due to the supportive and attentive nature of the teacher. This aligns with Gray et al. (19), highlighting the importance of unique skills and evidence-based knowledge among professionals throughout the system to effectively understand and meet these students’ needs.

It is well known that addressing SAP requires acknowledging the severity of students’ mental health issues and their lack of wellbeing at school. To address these mental health problems, the intervention needs to take a holistic approach to address the children's individual needs (2, 44). This study suggests that enhancing the knowledge and skills of professionals within the municipal support system is crucial for better supporting students with SAP.

A barrier highlighted by parents in this study is a lack of knowledge in schools and support systems. In line with previous research, professionals’ knowledge significantly influences municipal support systems’ efficacy in addressing SAP (12, 41). A deeper understanding of SAP is crucial, as attitudes and supportiveness are shaped by knowledge. Professionals need a comprehensive grasp of the multifaceted nature of SAP (23, 42). This allows for more effective identification of the root causes and will result in more targeted interventions (10).

The parents in this study stated that the knowledge held by the SAT should be implemented throughout the entire municipal system and the BUP to better support families and children with SAP. They said that only the SAT professional understood their situation thoroughly. They all valued the SAT's development of individualized support plans based on the results of the assessment phase in the HSP. The diverse roles of the SAT have positively impacted parents, providing them with a sense of relief and trust in their SAT professional. The parents often called the SAT professionals “our SAT professional.” However, one may speculate that this could also create a dependency that may prevent the development of sustainable relationships with the school and other professionals. This dependency between parents, students, and the SAT professional could lead to confusion and stress, making the system vulnerable. The complex role of the SAT professional in advocating for families can result in a sense of overload. Collaborating in teams with clear roles and responsibilities may help prevent such issues. HSP is one way to disseminate knowledge within a collaborative support system, with collaboration being a core element of the framework (15, 34). This may include continuous professional development to keep educators and professionals updated with the latest research, strategies, and practices in addressing absenteeism. Supporting students with school attendance problems requires collaboration not only between support systems but also in the SAT. Knowledge development should focus on intervention techniques, building strong relationships with students and their families, understanding diverse student needs, and fostering collaborative approaches (43). Further, based on the findings of this study, implementing the MTSS framework will meet some of the challenges by offering a structured and evidence-based approach to addressing absenteeism (46). Implementing this model could enable professionals to systematically evaluate and address the different levels of student needs (19). Within this multimodal approach, effectively addressing absenteeism requires combining it with individualized measures that are as unique as the students themselves (36). Furthermore, an ecological framework may be an effective way to increase competence and address SAP, supporting the implementation of a system that considers the organization and structure of the support and the importance of assessing all factors related to SAP (31). Reissner et al. (12) and Melvin et al. (25) show that a combination of a deeper understanding of absenteeism, the implementation of contextual frameworks, individualized and multimodal approaches, continuous professional development, fostering safe relationships, collaborative practices, and evidence-based interventions may significantly improve the effectiveness of support systems.

Multimodal strategies, which incorporate academic, behavioral, and social-emotional support, are essential in offering comprehensive intervention (5, 7, 23).

The results from this study showed that the evidence-based holistic approach used by the SAT provided effective interventions and collaboration for the diverse needs of students and their families. There is often a lack of coordination among the various professionals and services involved in addressing absenteeism. Valle et al. (17) underlined that a lack of coordination can lead to conflicting approaches, duplicated efforts, or gaps in support, making it difficult for parents to navigate the system and for students to receive cohesive and comprehensive care (17). Within an ecological framework, the system must establish a structure that promotes collaboration among the various systems. This will ensure that students are effectively directed to the appropriate services when encountering difficulties. Recognizing that absenteeism can be a symptom of psychiatric disorders, such as anxiety and depression, underscores the importance of collaboration within the support system (45). Such collaboration is crucial for the early detection and assessment of mental health issues and for preventing serious psychiatric problems and absenteeism (2, 43).

Professionals must be capable of building safe, trusting relationships with students and their parents. This level of support is a prerequisite to feeling supported and understood. A secure and trusting environment encourages open communication, which is crucial for identifying issues early and intervening effectively. Empowering parents will reduce the level of stress in both parents’ and students’ lives and increase the sense of wellbeing (17).

This study emphasizes the importance of collaborating with parents and students to ensure that all perspectives are considered in the decision-making process. These results support implementing an evidence-based, multimodal, multisystemic approach within the municipal support system (5, 14, 39). This approach should include systematic procedures within the support system and individually tailored measures to meet the needs of students with various levels of absenteeism (12–14). A holistic multisystemic approach could ensure that interventions are supported and effective (42).

## Conclusions and limitations

The results from this study highlight the importance of professionals’ knowledge and skills in providing evidence-based interventions for students and supporting parents. By deepening the understanding of absenteeism, adopting a multimodal approach such as HSP, engaging in continuous professional development, fostering safe relationships, promoting collaborative practices, and utilizing evidence-based interventions, the effectiveness of support systems can be significantly enhanced.

The study also identifies barriers, such as distrust, insufficient professional knowledge, systemic inconsistencies, and the undervaluation of parental involvement, which underscore the complexities of effectively supporting these students. The results of the study emphasize the importance of the SAT as a dedicated support unit equipped with the necessary knowledge, structures, and procedures to ensure that interventions are predictable, flexible, and transparent. To sustainably implement an SAT, municipalities must engage in informed discussions and provide the SAT with a mandate that reflects the complexity of the tasks and competencies needed to support children and their parents in this situation. This approach may ensure that interventions remain consistent over time and across various disciplines.

The valuable insights from this research will enrich existing literature and provide practical guidelines for policymakers and municipalities. Addressing parental perspectives gives important information that may improve support systems and ensure a more effective and higher-quality approach to addressing SAP.

A limitation of this study is the small sample size and diversity of the participants. The fact that most of the participants were mothers limits the diversity of the sample in this study, and this may represent a bias. The fathers’ voice is underrepresented and this may have given a one-sided presentation of the results. This may reduce the likelihood that the results represent all the parents who have experienced SAP and its challenges. One of the focus groups ended up with only two parents. This is not an ideal group size and may have affected the information gathered. The parents were eager to share their stories, and the results provided valuable information to the study, so the authors decided to conduct the interview session. The authors thoroughly discussed the study's validity during the analysis process.

Although the overall group size is small in this study, the transferability of the results to other contexts relies on a deep understanding of this specific context rather than generalizing to broader populations.

Another potential limitation of this study is that one of the authors previously served as a mentor for the SAT without being directly involved in supporting the parents. The parents were informed about this. The research group was one approach to minimize this effect. This bias was discussed thoroughly in the research group. Despite this, the results align with similar studies.

This study highlights the need for further research on parental support and collaboration within the system. Furthermore, the impact of integrating parents as equal partners in the support system and implementing multisystemic municipal approaches warrants further exploration. Further research is needed to establish a municipality support system based on evidence-based interventions.

## Data Availability

The original contributions presented in the study are included in the article/Supplementary Material, further inquiries can be directed to the corresponding author.
