# Correction: Parental perspectives on school attendance problems and the role of municipal support systems

**DOI:** 10.3389/frcha.2025.1645221

**Published:** 2025-06-27

**Authors:** Ulla Irene Hansen, Ellen Kathrine Munkhaugen, Kenneth Larsen

**Affiliations:** ^1^ Department of Education Science, University of South East of Norway, Horten, Norway; ^2^ Division of Mental Health and Addiction, Oslo University Hospital, Oslo, Norway

**Keywords:** parent's perspective, collaboration, empowerment, knowledge, school attendance problems

A Correction on Parental perspectives on school attendance problems and the role of municipal support systems By Hansen UI, Munkhaugen EK and Larsen K (2025). Front. Child Adolesc. Psychiatry 4:1589988. doi: 10.3389/frcha.2025.1589988

Affiliation “Department of Education Science, University of South East of Norway, Horten, Norway” was erroneously given as “Department of Psychology, University of Nevada, Las Vegas, NV, United States”. Affiliation “Division of Mental Health and Addiction, Oslo University Hospital, Oslo, Norway” was erroneously given as “Department of Psychiatry and Behavioral Sciences, Center for Developmental Epidemiology, Duke University Medical Center, Durham, NC, United States”.

The original version of this article has been updated.

